# Ensuring Population Health in the Era of Aging in Vietnam: Policy Review and Factors Associated with Intentions of Childbearing before the Age of 30 among Youths

**DOI:** 10.3390/healthcare11010102

**Published:** 2022-12-28

**Authors:** Linh Phuong Doan, Long Hoang Nguyen, Ha Ngoc Do, Tham Thi Nguyen, Giang Thu Vu, Hoa Thi Do, Carl A. Latkin, Roger C. M. Ho, Cyrus S. H. Ho

**Affiliations:** 1 Institute for Global Health Innovations, Duy Tan University, Da Nang 550000, Vietnam; 2 Faculty of Medicine, Duy Tan University, Da Nang 550000, Vietnam; 3 Department of Global Public Health, Karolinska Institutet, 17177 Stockholm, Sweden; 4 Ho Chi Minh Communist Youth Union, Youth Research Institute, Hanoi 100000, Vietnam; 5 National Centre for Youth Substance Use Research, The University of Queensland, Brisbane 4072, Australia; 6 Institute of Health Economics and Technology (iHEAT), Hanoi 100000, Vietnam; 7 Bloomberg School of Public Health, Johns Hopkins University, Baltimore, MD 21205, USA; 8 Department of Psychological Medicine, Yong Loo Lin School of Medicine, National University of Singapore, Singapore 119228, Singapore; 9 Institute for Health Innovation and Technology (iHealthtech), National University of Singapore, Singapore 119077, Singapore

**Keywords:** reproductive age, population, population policy, childbearing, delayed childbearing, low fertility rate, fertility intention, aging population

## Abstract

**Background:** Delayed childbearing has become a concern among policymakers across the world. In Vietnam, population policies have been adjusted to cope with this issue. In 2020, Decision 588/QD-TTg was ratified to encourage people to marry and give birth before the age of 30. This study reviewed recent changes in Vietnam’s population policies and assessed the intention of giving birth before 30 in young Vietnamese to provide insights into the potential effectiveness of the policy changes among young people. **Methods:** This study combined two approaches: a desk review and a survey of a group of youths. An online cross-sectional study was conducted on 116 respondents aged 16 to 30 in Vietnam from June to July 2020. The intention of childbearing before 30 and the importance and responsiveness of different social-environmental factors were asked using a structured questionnaire. We used multivariate logistic regression to identify associated factors of such intention. **Results:** The incentives set out by the Vietnamese government shared similarities with those of other countries. The plan of childbearing before the age of 30 among young adults in Vietnam was demonstrated to correlate with age, socioeconomic and biological characteristics, resources of the local health systems, as well as a clean and safe living environment. **Conclusion:** This study highlighted the recent crucial shift in Vietnam’s population policy. However, the quantitative analysis suggested that measures relating to environmental factors should be incorporated under this policy, implying that further interventions need to be taken into account to cope with delayed childbearing.

## 1. Introduction

The ages at which women bear children have significant implications for the world population’s future size and age structure [1]. For years, policymakers and scientists around the globe have recognized the importance of childbearing at the most fertile age in maintaining a population structure favorable for socioeconomic development, improving the quality of pregnancy, minimizing unwanted birth complications, consequently slowing population aging, reducing disease burden, and improving the overall quality of life. The scientific literature clearly states that the ideal range of maternal age to give birth is 20–30 years [2]. However, over the last decades, the number of women delaying childbearing until the third, even fourth decade of life has significantly risen in the developed countries [3] and become increasingly common in other regions, specifically the Asia-Pacific region [3,4,5]. The average age of first birth in Western countries ranged from 25 years in the United States (2006) to approximately 30 years in Switzerland (2010), with an increase of 2.9 years to 5.8 years since 1970 [3,5]. Countries such as Italy and the United Kingdom reported an even higher mean age of the first childbearing at around 31 years old [6,7]. Similarly, in the Asia-Pacific region, the mean age of the first childbearing varies from 28 in China and Malaysia to around 31 in Korea [8].

Marriage and childbearing postponement can have advantages. People have more time to gain financial stability and establish relationships, thus providing a more secure environment for their future children. Maturity and better parenting skill also come with older ages, and it has been evident that children born to older parents generally achieve higher levels of education [9]. With its advantages, delayed childbearing has been increasingly accepted globally [10]. Moreover, the literature review pointed out that difficulties in finding a suitable partner were one of the key reasons for delayed childbearing [11,12]. Nevertheless, health risks for the child and the mother at advanced ages outweigh the above benefits [13]. Birth in advanced age (≥35 years) could lead to various obstetric complications, including gestational diabetes, hypertension, preeclampsia, placenta previa, placental abruption, dysfunctional labor, cesarean delivery, postpartum hemorrhage, and maternal mortality, or fetal complications such as congenital anomalies, prematurity, growth restriction, macrosomia, and stillbirth [14,15,16,17,18,19,20]. A long-deferred first birth also increases the risk of chronic conditions such as breast cancer [21,22,23]. Besides risks to physical, advanced maternal age also affects physical health. In particular, some previous studies have reported that primiparous mothers at an advanced age (≥32 years of age) and a very advanced age (≥38 years of age) were more likely to experience depressive disorder and lower satisfaction with life during pregnancy compared to mothers aged 25–31 [24,25,26]. Changes in reproductive behavior have become a mutual concern among policymakers and scholars, especially in ultra-low fertility countries such as South Korea, Singapore, Hong Kong, and Japan [6,27]. Therefore, drastic policies and communication programs have been carried out to counteract with deferral of childbearing and low fertility rate [28].

In Vietnam, there has been a shift in fertility patterns during the last two decades, in which Vietnamese women tend to bear fewer children and give birth at older ages compared to 1999 [29]. Particularly, the mean age of Vietnamese women at first birth in 2011 was 30.1 years old, 6.3 years higher than in 1994 [8]. In 2020, the Vietnamese government ratified Decision 588/QD-TTg approving the birth rate adjustment program toward 2030 that encourages people to have their first child before 30, especially in cities and provinces with below-replacement fertility [30]. Most of these 21 regions are located in Southern Vietnam, the country’s critical economic region. Several research studies have been conducted on Vietnam’s population policies [31,32,33,34] which demonstrated success in reducing total fertility rate (TFR) to replacement level and slowing population growth rates. However, to our knowledge, no current work examines changes in Vietnam’s population policy since the country entered the aging population phase. In an attempt to fill the current lack of studies concerning Vietnam’s reproductive policy shifts and young people’s perception regarding childbearing age, this study is conducted to (a) document the evolution of Vietnam’s population policy, with a particular emphasis on the last decade to establish a rationale for recent policy changes, and (b) assess the intention to give birth before the age of 30 in young Vietnamese and identify factors associated with such intention.

## 2. Materials and Methods

This study used two approaches: (1) a desk review and (2) an online survey among a group of youths.

### 2.1. Desk Review

The desk review aimed to examine the population policy of Vietnam since the year 1961. By analyzing available population interventions, this study provided a general outlook on the evolution of Vietnamese population policy over periods, especially in each period’s political and socioeconomic context. Moreover, the review also highlighted the achieved results—both successes and limitations—to partly provide evidence on the formulation of the Decision 588/QD-TTg approving the birth rate adjustment program toward 2030. The peer-reviewed scientific literature on the effects of population policies was searched on PubMed, Medline, Google, and Google Scholar with different combinations of keywords, including “population intervention”, “population policy”, “developing country”, “Vietnam demographics”, etc. In addition, government reports, organizations’ reports, and legislative documents on population policy instruments in Vietnam were obtained and reviewed.

### 2.2. Study Setting and Participants

An online cross-sectional study was conducted from June to July 2020 in Vietnam. Using the snowball sampling technique, participants aged 16 to 30 (according to the definitions of youths in the Youth Law of Vietnam [35]) and living in Vietnam for at least six months were recruited from all provinces of Vietnam. The survey was first piloted on five youths to ensure its understandability and logic. The revision was then created on the online survey platform. We developed a core group including ten leaders of the Youth Union from different public institutions, companies, and organizations. After completing the survey, the participants invited their network peers to complete the online survey.

Regarding the sample size of this study, the formula for estimating a population proportion was applied with the expected proportion of people having their first child before 30 = 79% (according to previous research by Pew Research Center [36]), relative precision = 0.1, and confidence level = 95%. After calculating, the necessary sample size was 103 participants, and to prevent incomplete responses or dropout, 15% participant of the sample size was added, thus resulting in a total of 119 respondents being invited to be involved in this study. Finally, 116 participants agreed to participate in this study and complete the survey at the end of the data collection period, with a response rate of 97.5%.

The study protocol was granted by the institutional review board of Vietnam’s Youth Research Institute. We required electronic informed consent from participants. For those aged below 18 years, agreement and confirmation were obtained from their parents/guardians.

### 2.3. Measurement and Instrument

The questionnaire was created using SurveyMonkey’s platform (surveymonkey.com, accessed on 1 June 2020), consisting of three major components: (1) general demographic characteristics, (2) plan for childbearing before 30 years old, and (3) the assessment of conditions and responsiveness level of those related to such a plan.

#### 2.3.1. Socioeconomic Characteristic

We asked participants to report their information about gender, age, marital status (single/other), education (below high school and high school/college/tertiary and higher), and current living location (urban/town/rural or mountain area).

#### 2.3.2. Plan for Childbearing before 30 Years Old

One question, “Are you planning to bear a child before the age of 30?” with two options of the answer “Yes” or “No”, was used to assess the intention of respondents.

#### 2.3.3. Factors and the Responsiveness Level of the Factors Related to the Decision of Childbearing before 30 Years Old

Factors related to the childbearing decision before 30 years old: This questionnaire included 14 items presented in Table A1, describing the importance of different factors to the subject’s decision to give birth. For each item, the score ranged from 1 (not important) to 10 (very important), with a higher point indicating the greater importance of the factors.

The responsiveness level of factors relating to the decision of childbearing before 30 years old: This questionnaire included 14 items describing the responsiveness level of factors relating to childbearing before 30. For each item, the score ranged from 0 (totally non-responsive) to 10 (totally responsive), with a higher score indicating better responsiveness. The Cronbach’s alpha was 0.97.

#### 2.3.4. Data Analysis

The exploratory factor analysis (EFA) was used to restructure 14 items about the importance of conditions related to the decision of childbearing before 30 years old. The number of factors was determined with a threshold of the eigenvalue of 1.4 by scree tests. Items with a factor loading value ≥ 0.4 in an orthogonal varimax rotation with Kaiser normalization were considered included in the relevant component. The 2-factor model was optimal, with factor 1 that included 6 items (socioeconomic and biological characteristics of members in nuclear families) and factor 2 that included 8 items (social-environmental factors). The Cronbach’s alpha values were 0.93 and 0.92, respectively. The results of the EFA analysis are presented in Table A2. Descriptive and analytical statistics were conducted through the STATA software version 16. Multivariate logistic regression was used to identify factors associated with the desire for childbearing before 30 years old. Independent variables included: individual characteristics, conditions, and responsiveness levels. A *p*-value <0.05 was considered statistically significant.

## 3. Results

Population health refers to the health status and health outcomes within a group of people rather than considering the health of one person at a time [37]. These groups are often geographic populations such as nations or communities but can also be other groups such as employees, ethnic groups, disabled persons, pregnant women, or any other defined group. The health outcomes of such groups are affected by the policy in both the public and private sectors. Therefore, the work of assuring the population’s health experiences dramatic change, facing systemic problems and challenging societal norms and influences. In Vietnam, fertility patterns have changed during the last two decades, entering an aging population phase with one-third of cities/provinces having below-replacement fertility [37]. In this study, the shift in Vietnam’s population policy to adapt to each period was reviewed.

### 3.1. Evolution of Population Policy

Vietnam’s population policy has evolved from its inception in the 1960s to its legislation in the 2010s [34], which could be divided into five phases as follows (Table 1).

#### 3.1.1. 1961–1975

In 1960, the population of Vietnam was 30.2 million. On 26 December 1961, the Government Council issued Decision 216/CP on childbirth, making Vietnam one of the first Asian countries to issue official legislative documents and implement population and family planning programs. The primary goal of this Decision was to advocate for birth control by convincing people to participate in family planning to achieve the national goal of decreasing fertility. Population and family planning programs had achieved encouraging initial results. The population growth rate fell from 3,8% per year in 1960—which was extremely high as the result of high TFR—to 2,5% in 1975. The TFR also decreased from 6.3 children per woman in 1961 to 5.25 children per woman in 1975 [38].

#### 3.1.2. 1976–1990

Furthermore, 1976 was the first time that the population target was set out in the Resolution of the Fourth National Congress of Party as a growth rate of 2% by 1980 [39]. The next targets in the Fifth (1982) and Sixth Congress (1986) were 1.7% by 1985 and 1990 [40,41]. Since the inception of the Renovation Period (Doi Moi) in 1986, population policy was further developed with an emphasis on the promotion of one- to two-child families [34]. This phase also embarked on the implementation of the “reward—penalty” sanctions. Families with two children were provided subsidies for housing purposes. Three-child families were not allowed to register for households in cities, provinces, and concentrated industrial zones and had to pay more social security contributions. Birth control policies were extended in terms of target subjects and scope to all women and men of reproductive age across urban and rural areas. Birth planning policy was also associated with primary healthcare services, maternal and newborn health protection, and guidance on contraceptive methods for couples. As a result, the population growth rate decreased from 2.5% in 1975 to about 1.9% in 1990, and the TFR fell from 5.25 to 3.8 children per woman. While this result contributed to reducing the population growth rate, the set target was not achieved.

#### 3.1.3. 1991–2000

During this period, as a result of a rapid increase in population, reducing the population growth rate was of special interest to Vietnam and has become a national policy, described as “a large, vigorous and extensive movement among the entire people” [42]. In 1993, the 7th Party Central Committee Conference issued Resolution 4 on population and family planning which was the first formalization of the one- to two-child policy later [34]. Following this Resolution, the first National Strategy on Population and Family Planning 1993–2000 was launched by the government, with the key objective of reducing TFR to 2.9 by 2000 (NCPFP, 1993). The population policy in this stage shared similarities with the promotion of one- to two-child families in the previous stage, focusing on diversifying family planning services. The “reward-penalty” policy maintained since the period of 1976–1990 was also applied more drastically. The population program peaked in the 1990s [34], contributing to a dramatic drop in TFR from 3.8 in 1991 to 2.3 in 2000, 0.6 lower than the target of 2.9. Other important set targets were exceeded: The population size increased from 67.2 million people in 1991 to 77.6 million people in 2000, lower than the target of about 82 million by 4.4 million people. The population growth rate decreased from 1.86% (1991) to 1.36% (2000), suggesting that rapid population growth has been restrained [38].

#### 3.1.4. 2001–2010

Significant changes in the priority objectives of population policy were made in these years, marking major milestones in the development of the population policy of Vietnam associated with the implementation of the National Strategy of Population in the period of 2001–2010. In 2006, the TFR of Vietnam was 2.09, achieving the replacement fertility rate ten years earlier than the target set out in 1993 in Resolution 4-NQ/TW. The priorities of population policy had shifted from “controlling population size” to “improving population health,” and the content of population size changed from “actively controlling” to “actively adjusting”. Population growth was no longer considered an obstacle but rather a driving force for the socioeconomic development of the country. As a result, Vietnam has encouraged voluntary childbearing since 2003. The “reward—penalty” sanctions were applied to a certain extent to specific social groups.

The Population Ordinance 2003 was issued by the Standing Parliamentary Committee of the National Assembly in 2003, serving as the highest-ever legislative document of population policy. The Decree prohibition obstructing or forcing the implementation of family planning and fetal sex selection was issued by the government, ending a period of application of coercion and shifting to encouraging people’s voluntariness of family planning. Notably, for the first time, reproductive rights were legally acknowledged in Article 10 of the Ordinance 2003, which stated that couples had the right to decide the number of children they wanted, as well as the timing and spacing of their births [43]. This resulted in misinterpreting that the state encourages large families, causing TFR and the third childbirth rate to increase sharply again. Therefore, the population growth rate increased dramatically in the next three years. In 2009, the population sector had a more specific message that changed to “Each couple should have two children”. In general, the fertility rate decreased by 1.5% in the period 2006–2010. The population growth rate was 1.05% in 2010. The population size in 2010 was 86.92 million people, lower than the forecast made in 1990 of 18.5 million people.

#### 3.1.5. 2011–Present

In 2011, population aging commenced in Vietnam. Other emerging challenges in terms of employment, accommodation, social welfare, shortage of trained workers, imbalance in the sex ratio at birth, disparities between urban and rural areas, etc., had hampered Vietnam’s ability to seize its demographic opportunity. Vietnam’s Strategy on Population and Reproductive Health in the period 2011–2020 emphasized reproductive healthcare, avoiding unwanted pregnancy in adolescence, strengthening antenatal and neonatal screening, promoting gender equality, and sustainable development of the country [44]. In January 2016, the Party’s Conclusion affirmed a focus shift from family planning to population development. This shift is aimed at comprehensively solving population problems regarding size, structure, and distribution and improving population health [45].

The average annual population growth rate in the period 2009–2019 was 1.14 percent per year. Vietnam has maintained a stable TFR at replacement level for more than a decade; the perception of having two children in Vietnam has become a social norm. However, the average birth rate differs significantly across the country depending on different localities and regions, socioeconomic development, education, living standards, etc. The Northern Midlands and Mountains, the Red River Delta, the North Central Coast, the South Central Coast, and the Central Highlands were regions with above-replacement fertility rates, while the two regions with sub-replacement rates were the Southeast and the Mekong Delta. Specifically, Ho Chi Minh City—the biggest city and an economic hub of Vietnam—has the lowest fertility rate of 1.39 children per woman of reproductive age [29].

On 28 April 2020, Prime Minister Nguyen Xuan Phuc inked Decision 588/QD-TTg approving the fertility rate adjustment program toward 2030. The program has set the target of maintaining a replacement fertility rate of between 2 and 2.2 per woman in reproductive age, decreasing the TFR in high-fertility areas while boosting it in low-fertility areas, aiming at addressing a falling fertility rate and coping with demographic challenges arising from the aging population [30]. It was noteworthy that the Decision has encouraged people to get married before 30 years old and have children early, preferably before 35, which would be applied only to low fertility rate areas to address the issue of delays in getting married and having children among the youths. Along with this encouragement, the regulations involving the fertility reduction goal would be amended and relaxed, whereas the policy to encourage the birth of two children per family would be further promoted.

Under Decision 588, several incentives for couples having two children would be offered in a pilot project in localities with a low birth rate: providing maternal and child healthcare and counseling, including infertility screening, prenatal and postnatal screening, and malnutrition prevention; creating favorable conditions for women upon return to the workplace after maternity leave; and offering a personal income tax rebate, exemption, or reduction of the household’s public fees. They would also have an allowance for purchasing or renting social housing, priority in public school registration, and financial support for tuition fees. Other support policies have been taken into account: providing marriage counseling and services such as dating clubs and premarital health counseling; creating a family-friendly environment and community through developing services such as babysitting, breast milk banking, and family physicians; and sufficiently providing nursery facilities and kindergartens, especially in urban and industrial areas. Additionally, measures to increase responsibility for social and community contributions to individuals who do not wish to marry or marry too late would also be piloted. Following that, an evaluation would be conducted in order to establish an official policy.

After the launch of Decision 588, some policies have been adjusted and piloted in some areas [46]. Particularly, according to the Department of Population and Family Planning, in regions with low fertility rates, regulations of organizations, agencies, units, and communities related to fertility reduction goals and criteria for no more than two children have been abolished [47]. For example, in the past, many localities often set criteria on population and family planning that no family gave birth to a third child or more. After Decision 588, these criteria were removed to raise fertility, aiming to maintain the replacement fertility rate. Furthermore, in regions with low fertility rates, measures to support couples to have two children, such as marriage and family services, personal income tax reduction, support to purchase housing/rent house, giving priority to public schools, and support to children’s education expenses, have been piloted [48].

### 3.2. Preference of Youths toward Birth before 30

Overall, the percentage of participants having a plan of childbearing before 30 was 61.2%. The mean scores of socioeconomic and biological characteristics and social-environmental factors were 8.8 ± 1.9 and 8.1 ± 2.2, respectively. Safe living environment (8.1 ± 2.7) and clean living environment (8.0 ± 2.7) had the highest response level, while opinions of parents and relatives on childbearing before 30 (6.8 ± 3.6) and pregnancy process (6.8 ± 3.8) had the lowest response. In terms of associated factors, higher scores of socioeconomic and biological characteristics (OR = 1.86; 95%CI: 1.01; 3.42) and a clean living environment (OR = 2.61; 95%CI: 1.09; 6.26) increased the likelihood of childbearing intention before 30. By contrast, a safer living environment was associated with a lower likelihood of childbearing before 30 (OR = 0.27; 95%CI: 0.0.10; 0.73) (Table 2).

## 4. Discussion

### 4.1. Policy Analysis

Vietnam’s population policy has developed from pursuing a major objective of fertility rate reduction in the last century to covering a wide scope of objectives relating to family planning, population health, enhancement of social welfare, education and basic healthcare, etc. Although the shift in policy was initiated in 2016 when Resolution 47-NQ/TW was issued, Decision 588/QD-TTg represented a landmark in the development of the policy, with the objectives shifted from decreasing the fertility rate to maintaining the replacement level to cope with the aging population.

Generally, the effectiveness of and the population’s reaction to Vietnam’s policies are different from Western countries due to its socialist regime. The Vietnamese political system is authoritarian, meaning new regulations or policies are generally met with compliance. Indeed, a high level of compliance with new laws among Vietnamese has been evident in numerous circumstances, most recently in the COVID-19 pandemic [49]. In the context of childbearing, new directives had profound impacts on citizens’ perception and decision-making process of childbearing, as the patterns in population rate at any given period fluctuated in accordance with current regulations and national goals.

Besides political compliance, several incentives were provided for the Vietnamese population to follow the set-out goal. Decision 588/QD-TTg incorporated the same three categories of incentives that vary across countries, including financial incentives, support for parents to combine work and family, and broad social change supportive of children and parenting [50]. Guidelines in Decision 588 were identical to those of other countries’ programs such as Singapore. Since the 1980s, Singapore has launched a program encouraging the birth of a third child to counteract the extremely low fertility rate. The incentives included tax rebates, priority in housing for the family, and school registration for children. Additionally, the Singaporean government has established a unit to foster interaction among male and female university graduates [51]. While the above financial incentives could bring about short-term effects, other sustainable solutions, such as developing a family- and child-friendly environment or changing couples’ perceptions of late childbirth, would take more time. However, it is not easy to engage employers to create favorable conditions for employees to fully enjoy allowed maternity or childcare leave. Governmental leadership is essential to coordinate government, employers, and families on a broad scale [50]. This is also an important suggestion for the future comprehensive implementation of Decision 588′s policy guidelines in Vietnam.

The policy in effect for the longest duration was the two-child policy, which was an adaptation from China’s strict one-child policy from 1980 to 2016. This policy has apparent advantages and effects, such as a slowdown of population growth, secured livelihood, education quality, and better allocation of resources, to name a few [49,52]. In the longer term, however, this policy is detrimental to the nation’s workforce as it results in the loss of human resources. Moreover, previous implementations demonstrated that coercion for citizens to bear a certain number of children may be counter-productive and can lead to demographic distortion, such as in China [53].

Realities have shown that a number of nations have succeeded in lowering their birth rates. On the other hand, there is no successful model for increasing fertility once it has fallen too low [50]. It is a long-term challenge for Vietnam in terms of increasing fertility in low-fertility areas and tackling the deferral of childbearing. The implementation of Decision 588 requires additional research on fertility behaviors in order to develop conducive particular interventions.

### 4.2. Quantitative Analysis

The survey of 116 young people provided insights on the potential effectiveness of the policy changes among young people, as well as suggested further interventions from the youth perspective. In particular, our study indicated a moderate rate of young people planning to give birth before the age of 30 (61.2%). Additionally, the study showed that the intention of childbearing before 30 was related to age, socioeconomic and biological characteristics, resources of the local health system, and a safe and clean living environment, suggesting further supportive policies and interventions to promote childbearing before 30 among young adults in Vietnam.

The findings of this study indicated that 61.2 percent of young adults in our sample reported the intention of childbearing before the age of 30, which was higher in comparison to that previously reported in England (50.1%) [54], and lower than that in the United States (79%) [36], suggesting that Vietnamese youth attitudes and perceptions have shifted in recent years toward traditional conception viewing marriage and childbirth as essential for women at the age of 20.

Previous reports revealed that a clean living environment is a condition of the air, water, land, and energy that is free of unwanted matter and able to affect human health [55], and a safe living environment is one in which the population has the freedom to pursue daily activities without fear of violence, an adequate level of public order, and accountable security forces to protect key individuals and communities, as well as the freedom for people [56]. These conditions are essential for human existence as well as direct effects on decisions in human life, including childbearing decisions. In our study, the regression results revealed that the safer living environment was negatively associated with a likelihood of planning childbearing before the age of 30. This finding could be explained in part by the fact that people were more satisfied living in a safer environment and could enjoy their life with partners, friends, work, and entertainment without having to worry about children. Additional qualitative research is required to explore this issue further. On the other hand, a cleaner living environment would increase the likelihood of planning childbearing before 30. Environmental factors are closely linked to not only prenatal and postpartum health but also children’s quality of life and later development [57,58]. Adverse pregnancy outcomes relating to environmental factors, including congenital anomalies, increased risk for miscarriage, preterm delivery, intrauterine growth restriction, and stillbirth, may occur. However, rapid technological development in pregnancy care has made people underestimate the above risks because of the belief in the success of assisted reproductive technologies. Egg freezing services, for instance, have become the choice of more and more women who want to delay childbearing to pursue academic or professional careers despite the fact that it carries risks to physical and mental health [59,60]. Moreover, the recent increase in the privatization of healthcare delivery has also resulted in a better quality of prenatal care [61]. Additionally, improved awareness and accessibility to healthcare services, as well as enhanced quality of pregnancy care, have contributed to easing the concerns about the risk of complications for the child. Hence, these concerns may not be a primary factor affecting the intention of childbearing among Vietnamese women. However, the ability to access reproductive healthcare services may be significantly influenced by socioeconomic and cultural factors, subsequently leading to the risk of inequities in healthcare which could affect the decision to have children in certain areas [62,63]. In addition to the concerns about adverse outcomes during pregnancy, the medicalization of childbirth and the use of controversial methods such as episiotomy and uterine fundal pressure in women during delivery can also cause dangers and complications for the mother in the long term [64,65].

This study informed several important implications. First, the government should launch information, education, and communication campaigns to raise awareness of the benefits of childbearing and childrearing before 30, as well as the adversity of delayed childbearing, with target audiences being both male and female. Second, in terms of the environment, policies should be implemented to foster environmental movements in the neighborhood, enabling everyone to contribute to the creation of a child-friendly and clean environment. Last but not least, it is critical to increase supportive policies for female employees on maternity leave and take measures to assist them upon their return to work. Simultaneously, supportive policies should be expanded to cover male employees whose wives become pregnant. This would enable husbands to further support their wives, relieving the great burden of childcare on women and providing them with psychological stability during the postpartum period.

In comparison to the incentives set forth in Decision 588/QD-TTg approving the fertility rate adjustment program toward 2030, the policy implications from this survey’s results shared similarities. The Decision proposes three groups of measures: (1) education and communications for behavior change, emphasizing the advantages of having two children, the disadvantages of late marriage and childbirth for individuals and the country’s socioeconomic development, and diversifying the methods and forms of campaigns; (2) incentive programs from central to grass-root levels, including financial incentives, counseling services, and creating a family-friendly community; and (3) expansion of access to reproductive healthcare and related services. By and large, these measures are relatively comprehensive and feasible, with concentrations on a variety of aspects. However, environmental factors have been omitted from this legislative document, even though they are significant determinants of young people’s decision to have children. In addition, supportive policies for females have been neglected. In the future, these interventions should be included to meet the demands of people for antenatal care and childcare and raising.

### 4.3. Limitations

This study had limitations that should be noted. Firstly, due to the sample selection with purposes, its generalization ability was limited. Additional groups of young people with distinct individual characteristics, such as particular living environments, special types of jobs and occupations, or fields of study, were not included in the survey, which might be important confounding factors. Hence, future research should include these variables in order to obtain more comprehensive results. Secondly, the average participant age was approximately twenty years old, with more than 90% unmarried. Other groups of samples with a higher mean age or who already have children might produce different results. However, the study’s findings still provided insights into the youths toward childbearing before 30, thereby suggesting prompt interventions for policymakers, especially in the early stage of implementing the Vietnam Strategy on Population and Reproductive Health in the period 2011–2020.

## 5. Conclusions

This study highlighted the recent crucial shift in the development of Vietnam’s population policy in which Decision 588 in 2020 was formulated, encouraging young males and females in 21/63 cities and provinces with low TFR in Vietnam to tie the knot and bear a child before 30 years old. Simultaneously, the plan of childbearing before the age of 30 among young adults was demonstrated to correlate with age, socioeconomic and biological characteristics, resources of the local health systems, and a clean and safe living environment. Although comprehensive incentives relating to these associating factors were incorporated under Decision 588, the environmental factors and incentives for females need to be taken into account in future interventions to cope with delayed childbearing.

## Figures and Tables

**Table 1:** Evolution of population policy in Vietnam, 1961–2020.

Period	Year	Legislative Documents	Objectives
1961–1975	1961	Decision 216-HDBT	Establishing the Population and Birth Control Unit (1961–1983)
	1961	Decision 216/CP on guided childbirth	Promoting birth control
	1963	Directive No. 99/TTg on providing prenatal guidance	
	1970	Decision No. 94/QD on campaigning for planned births	
1976–1990	1976	The Resolution of the IV National Party Congress	Striving for a population growth rate of 2% by 1980
	1978	Directive No. 265/CP on promoting birth control throughout the country	
	1981	Directive No. 29/HDBT on promoting birth control for the period of five years, 1981–1985	
	1982	The Resolution of the V National Party Congress	Reducing the country’s average annual population growth rate from 2.4% to 1.7% by 1985
	1984	Decision 58-HDBT	Establishing the National Committee on Population and Birth Control (1984–2000)
	1986	The Resolution of the VI National Party Congress	(1) Reducing the population growth rate from 2.2% to 1.7% by 1990 (2) Simultaneously implementing measures such as investing in the construction of community-level healthcare facilities and centers to assist women during childbirth
	1988	Decree 162-HDBT	Regulating population and birth control policy
1991–2000	1992	The Prime Minister promulgated the Decision on the Strategy of population and family planning communication	
	1993	Resolution 4-NQ/TW on population and family planning programs were adopted at the Seventh National Party Congress	
	1993	Decision 270-TTg approving the National Strategy on Population and Family Planning 1993–2000	
	1995	Directive 50-CT/TW on strengthening the implementation of the population and family planning program	
	1997	Decision 37-TTg on accelerating the implementation of the National Strategy on Population and Family Planning 1993–2000	
2001–2010	2001	Decision 72/2001/QD-UB	Establishing Vietnam Commission for Population, Family, and Children (2001–2006)
	2001	Launch of National Strategy on Population 2001–2010	Improving the population’s quality of life, developing high-quality human resources to meet industrialization and modernization needs, and contributing to the country’s rapid and sustainable development
	2003	The Population Ordinance	Stating that couples had the right to decide the number of children they want, as well as the timing and spacing of their births
	2003	Decree on the prohibition of obstructing or forcing the implementation of family planning and fetal sex selection	
	2005	Resolution 47-NQ/TW issued to reinforce the birth control policy	(1) Actively adjusting the population’s size and quality in accordance with the requirements of socioeconomic development (2) Enhancing the quality of reproductive healthcare: family planning services (3) Utilizing the correlation between rational population distribution and population management and human resource development
	2008	Decision 18/2008/QĐ-TTg	Establishing the General Office for Population and Family Planning (2008-present)
	2008	The revised Population Ordinance approved	
	2010	Decree ND-CP 20	Guidelines for the implementation of the revised Population Ordinance
2011–2020	2011	Decision 2013/QD-TTg approving Vietnam Strategy on Population and Reproductive Health in the period 2011–2020	(1) Enhancing population health (2) Improving reproductive health status (3) Maintaining a reasonably low fertility rate (4) Solving problems of population structure and distribution
	2016	Conclusion No. 119-KL/TW on the continuation of the implementation of IX Politburo Resolution No. 47-NQ/TW (2005)	(1) Reducing fertility in provinces and cities with high fertility rates (2) Maintaining results achieved in provinces and cities where fertility has reached the replacement level (3) In areas with low fertility, each couple should have two children
	2017	Resolution No. 21-NQ/TW on population in the new situation	Continuing to shift the focus of population policy from family planning to population and development
	2019	Decision 1679/QĐ-TTg Approving Vietnam Population Strategy toward 2030	Firmly maintaining the replacement fertility rate; bringing the sex ratio at birth to the natural balance; making full use of the demographic window of opportunity; adapting to the aging population; rationally distributing the population and improving population health; and contributing to the rapid and sustainable development of the country
	2020	Decision 588/QD-TTg approving the fertility rate adjustment program toward 2030	Increasing the TFR by 10% in provinces and cities with low fertility rate (<2 children/woman), reducing the TFR by 10% in the high-fertility areas (>2.2 children/woman), and maintaining the replacement fertility rate in the regions which have reached the level (from 2 to 2.2 children/woman)

**Table 2:** Multivariate logistic regression to identify factors associated with plans of childbearing before 30 years old among Vietnamese youth (n = 116).

Characteristics	Total		Plan to Childbearing before 30	
	n	%	OR	95%CI
**Total**	116	100		
**INDIVIDUAL CHARACTERISTICS**				
**Age (per year), Mean (SD)**	19.9	3.1	1.19	0.97; 1.47
**Marital status**				
Other	10	8.6	Ref.	
Single	106	91.4	0.51	0.07; 3.67
**Current living**				
Urban	61	52.6	Ref.	
Suburban	20	17.2	1.13	0.28; 4.67
Rural	35	30.2	0.88	0.30; 2.60
**Gender**				
Male	35	30.2	Ref.	
Female	81	69.8	2.04	0.72; 5.80
**Education**				
Below high school or high school	41	35.3	Ref.	
College	21	18.1	0.8	0.15; 4.14
Under or postgraduate	54	46.6	0.69	0.19; 2.51
**Plan to childbearing before 30**				
No	45	38.8		
Yes	71	61.2		
**CONDITION AND RESPONSE LEVEL**	Mean	SD		
**The importance of conditions related to the decision of childbearing before 30 years old**				
Socioeconomic and biological (range: 0–10), (unit: per score)	8.8	1.9	1.86 **	1.01; 3.42
Social-environmental factors (range: 0–10), (unit: per score)	8.1	2.2	0.82	0.48; 1.41
**The response level of the conditions related to the decision of childbearing before 30 years old (range: 0–10), (unit: per score)**				
Safe living environment	8.1	2.7	0.27 ***	0.10; 0.73
Clean living environment	8	2.7	2.61 **	1.09; 6.26
The health of myself and my lover/spouse	7.8	3.1	0.91	0.68; 1.23
Resources of the local health system	7.6	3	1.60	0.95; 2.69
Government-supportive policies for pregnant women	7.5	3	0.96	0.56; 1.66
Resources of the local educational system	7.5	3.2	0.91	0.60; 1.35
Workplace supportive policies for pregnant women	7.3	3.2	0.83	0.50; 1.39
The health of the child at birth	7.2	3.6	1.11	0.73; 1.69
The ability to raise children for myself and my lover/spouse	7.2	3.6	1.02	0.67; 1.56
Friends and relatives having children before 30	7	3.5	1.27	0.92; 1.76
Time of myself/lover/spouse	6.9	3.5	0.86	0.68; 1.09
The economic ability of myself and my spouse	6.9	3.6	0.94	0.61; 1.43
Pregnancy process	6.8	3.8	1.17	0.86; 1.59
Opinions of parents and relatives on childbearing before 30	6.8	3.6	0.93	0.66; 1.30

*** *p* < 0.01, ** *p* < 0.05.

## Data Availability

The raw data supporting the conclusions of this article will be made available by the authors without undue reservation.

## Appendix A

**Table A1:** Assessment of the importance of factors related to the decision of childbearing before 30 years old among youths (n = 116).

Items (Range: 0–10)	Very Important		Factor 1: Socioeconomic and Biological Characteristics	Factor 2: Social-Environmental Characteristics
	n	%		
The health of the child at birth	88	75.9	0.88	
The ability to raise children of myself and my lover/spouse	81	69.8	0.89	
Safe living environment	81	69.8		0.91
Pregnancy process	76	65.5	0.83	
Clean living environment	76	65.5		0.90
The economic ability of myself and my spouse	73	62.9	0.91	
Health of myself and my lover/spouse	71	61.2	0.81	
Response of the health system where you live	62	53.5		0.65
Workplace supportive policies for pregnant women	62	53.5		0.67
Government-supportive policies for pregnant women	61	52.6		0.62
Response of the educational system where you live	59	50.9		0.67
Time of myself/lover/spouse	46	39.7	0.54	
Friends and relatives have children before 30	44	37.9		0.84
Opinions of parents and relatives on childbearing before 30	42	36.2		0.84
**Cronbach’s alpha**			0.93	0.92
**Mean SD**			8.8	8.1
**SD**			1.9	2.2

**Table A2:** Scores of condition assessment for childbearing before 30 years old decision among Vietnamese youth (n = 116).

	Male		Female		Total		*p*-Value
	Mean	SD	Mean	SD	Mean	SD	
**Socioeconomic and biological characteristics of members in a nuclear family.**							
The health of the child at birth	8.4	2.9	9.6	0.9	9.3	1.8	0.04
The ability to raise children of myself and my lover/spouse	8.3	2.9	9.5	1.1	9.1	1.9	0.04
The economic ability of myself and my spouse	8.2	2.9	9.4	1.1	9.0	1.9	0.09
Pregnancy process	8.2	3.1	9.2	1.4	8.9	2.1	0.34
Health of myself and my lover/spouse	7.7	3.3	9.2	1.4	8.7	2.3	0.11
Time of myself/lover/spouse	7.2	3.6	8.0	2.4	7.8	2.8	0.79
**Social-environmental factors**							
Clean living environment	7.9	3.0	9.4	1.1	9.0	2.0	0.01
Safe living environment	8.1	2.9	9.5	1.2	9.0	2.0	0.03
Workplace supportive policies for pregnant women	7.7	3.0	9.0	1.5	8.6	2.1	0.04
Government-supportive policies for pregnant women	7.6	3.1	8.8	1.6	8.5	2.2	0.08
Resources of the local educational system	7.8	3.0	8.6	2.2	8.4	2.5	0.25
Resources of the local health system	7.6	3.1	8.5	2.2	8.2	2.5	0.28
Opinions of parents and relatives on childbearing before 30	7.3	3.3	6.9	3.4	7.0	3.3	0.48
Friends and relatives have children before the age of 30	7.2	3.4	6.8	3.5	6.9	3.5	0.47
